# Revisiting an Underrecognized Strategy for Rhythm Management: Hybrid Therapy for Patients who Convert from Atrial Fibrillation to Flutter on Antiarrhythmic Drugs

**DOI:** 10.19102/icrm.2019.101005

**Published:** 2019-10-15

**Authors:** Fady S. Riad, Albert L. Waldo

**Affiliations:** ^1^Division of Cardiovascular Medicine, University Hospitals Cleveland Medical Center, Cleveland, OH, USA

**Keywords:** Antiarrhythmic drugs, atrial fibrillation, atrial flutter, catheter ablation, class Ic antiarrhythmics

## Abstract

Atrial fibrillation (AF) is often treated with antiarrhythmic drugs (AADs) or catheter ablation. In a unique subset of patients, AF can convert to atrial flutter (AFL) after the initiation of an AAD. It has previously been shown that, in this subset of patients, cavotricuspid isthmus (CTI) ablation followed by the continuation of the AAD regimen has an unusually high rate of successfully maintaining sinus rhythm. This is an underrecognized approach toward rhythm management in such patients. However, the reason(s) for such a high degree of efficacy with this hybrid therapeutic approach are unclear. We suggest that conversion from AF to AFL selects for a group of patients in whom AF is particularly responsive to the effects of the AAD. Since CTI ablation is essentially curative of AFL, the combination of both techniques results in a high efficacy of sinus rhythm maintenance. Further investigation is required to confirm these hypotheses.

## Introduction

Patients with atrial fibrillation (AF) are most often treated with antiarrhythmic drugs (AADs) as a first-line strategy for rhythm control. As is well-documented, such treatment results in varying degrees of efficacy. Importantly, a subset of these patients will convert from AF to atrial flutter (AFL). It has been shown that, in patients treated with a class IC antiarrhythmic drug (AAD), as many as 20% will convert from AF to typical AFL.^1^ After successful cavotricuspid isthmus (CTI) ablation and, with continued administration of their AAD, a remarkably high number of these patients will maintain sinus rhythm. Several small studies in the late 1990s to early 2000s evaluated patients with paroxysmal or chronic AF that converted to AFL after receiving an AAD—usually flecainide, another class IC agent, or amiodarone—and assessed arrhythmia-free survival after CTI ablation along with maintaining therapy with the AAD. Their results showed remarkable efficacy for the long-term maintenance of sinus rhythm, including rates of up to 80% to 90% in some. Others showed more modest results for arrhythmia-free survival, but all highlighted a substantial decrease in symptomatic recurrence and frequency of recurrence **(Table 1)**.^2–6^ This strategy, however, is underrecognized in current clinical practice, and no large prospective trials of this method of treatment exist at this time. Nevertheless, we suggest that consideration of this approach for treating AF, particularly in patients with new-onset or paroxysmal AF, should be given as long as there are no contraindications to using a class Ic antiarrhythmic in a particular case. Finally, the reasons for the unique effectiveness of this hybrid approach to treating AF are unknown. What follows thus is our attempt to understand the whys and wherefores of this unique mode of therapy.

## Role of class Ic antiarrhythmics for the restoration of sinus rhythm in the general population

Flecainide is an effective medication for the cardioversion of AF, resulting in a 70% to 90% rate of conversion to sinus rhythm within eight hours of administration.^7–13^ It has also been shown to significantly reduce the recurrence of AF as well as prolong the time to first AF recurrence and the time interval between AF recurrences.^14–17^ The rate of AF recurrence within several months to one year has been shown to be between 30% and 70% among various studies, although, in patients deemed “difficult to treat,” the effect was predominantly an increase in the time between AF recurrences, with no significant impact on total burden of AF recurrence.^15^ Propafenone, another class Ic agent, has been shown to have similar efficacy.^12,13,18,19^ This class of agents, however, has been associated with seemingly “proarrhythmic” effects—namely, conversion to a relatively slow AFL—that, when slow enough, may result in 1:1 atrioventricular (AV) conduction in the absence of sufficient AV nodal blockade. The exact incidence of this phenomenon is unclear, as most reports are in the form of case reports or small case series.^20–22^ Fortunately, this symptomatic conversion is rare and the adequate use of AV node–blocking agents should prevent its occurrence.^1,2,23^

## A hybrid approach to rhythm management in patients with atrial fibrillation that converts to typical atrial flutter on class Ic agents

Some have suggested this “proarrhythmic” effect of AADs (ie, conversion of AF to AFL) to be less of an obstacle and more so an opportunity, hypothesizing that, when such patients converted from AF to typical AFL, the addition of CTI ablation would not only address the treatment of AFL but also enhance the suppression of AF. As noted above, this clinical approach demonstrated a very high rate of efficacy when AF converted to typical AFL.^2–6^

Understanding these clinical outcomes, however, requires comprehension of the relationship between AF and typical AFL. This relationship has long been documented in the literature.^24,25^ Specifically, it has been noted that AFL rarely forms without a preceding episode of AF.^26^ Such AF episodes are often underrecognized because they are typically short, lasting only seconds to minutes.^27^ These observations are bolstered by the finding that, in patients with concurrent AF and typical AFL, successful pulmonary vein isolation (PVI) alone was as effective as PVI plus CTI ablation for the prevention of AFL recurrence.^28^ This finding supports the notion that AF is the trigger for AFL, since elimination of the AF by PVI alone resulted in elimination of the AFL as well.

## Physiology of the development of atrial flutter

**Figure 1:**
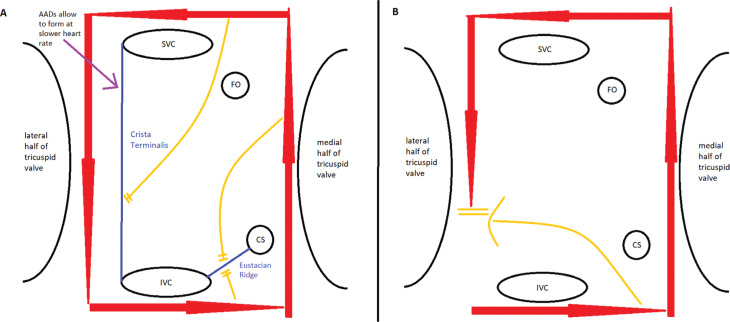
View of the right atrium from a bisected tricuspid valve toward the posterior wall. **A:** AFL circuit (red arrows) traversing in a counterclockwise direction around the tricuspid valve. Functional lines of block primarily along the crista terminalis and also the eustachian ridge (blue lines) prevent short-circuiting of the circuit (yellow lines). AADs promote the formation of these lines by reducing the threshold rate for formation to below the rate of the AFL circuit. **B:** AFL circuit (red arrows) traversing in a counterclockwise direction around the tricuspid valve. The lack of a line of block between the venae cavae allows for short-circuiting of the circuit around the superior vena cava or inferior vena cava (yellow arrow), causing termination of the macroreentrant circuit.

Another important aspect of the development of typical AFL is the formation of a functional line of block in the right atrial free wall between the venae cavae, which is necessary to prevent short-circuiting of the macroreentrant AFL circuit. This was initially demonstrated in a canine model, which showed that, in all episodes of AFL, such a line of functional block was developed.^29^ Furthermore, it was demonstrated that, when this line of block decreased in length to a critical value, the AFL converted to AF, consistent with the notion that this line of block was critical for the maintenance of typical AFL **(Figure 1)**. These findings have been confirmed through invasive electrophysiologic studies of AF in humans and the line of block was localized to the crista terminalis.^30–32^ Notably, it was shown that this line of block developed in a rate-dependent manner, with shorter AF cycle lengths being more likely to result in conduction block.^32,33^ Importantly, the cycle length required to maintain the line of block in patients with AF was significantly shorter than that in patients with AFL.^33,34^ This finding is critical because if the rate required to maintain the line of block was significantly faster than the rate of the resulting AFL circuit, the line of block could not be maintained and the reentrant circuit would degenerate into AF once more. This concept of the importance of intercaval block is supported by findings that patients after the surgical repair of an atrial septal defect often present with AFL, not AF.^35,36^ We suggest that this occurs because the fixed line of intercaval block from the surgical scar is present to maintain the reentrant circuit. Finally, it has been shown that antiarrhythmic therapy with amiodarone can also promote the development of this line of block, associated with the development of AFL.^34^

Synthesis of the above findings suggests that the development of typical AFL begins with an episode of AF that, due to rapid atrial rates, leads to the onset of a functional line of block along the crista terminalis (essentially a block between the superior and inferior venae cavae). In the right patient, the rate required for formation of this functional block is slow enough that, once the AFL circuit forms, it is able to sustain the line of block on its own. Antiarrhythmics, especially those that slow conduction through the atrial myocardium or increase the atrial refractory period, may decrease the threshold for the formation of this line of block and, therefore, promote the conversion of AF to AFL. There is additionally some evidence that short delays in electrical activity in the right atrial free wall are ubiquitously seen immediately preceding the conversion of AF to AFL.^27^ This period potentially allows for or is a result of the organization of the various activation wavefronts in AF and enables the formation of a single wavefront that becomes the AFL reentrant circuit. Although this finding requires further confirmation, it could be the final piece of evidence needed to explain why some patients convert to AFL and others do not. Essentially, once the requisite conditions for the formation of AFL are obtained, there is a component of chance that the multiple wavefronts during AF will interact in such a way that they form a functional intercaval line of block as well as a unified wavefront that will circulate around it and form the AFL reentrant circuit. Statistically, this would occur sooner or later and would primarily be a function of time.

## Role of class Ic agents in the hybrid strategy for rhythm management

Notably, while an understanding of the above can explain why we see conversion from AF to AFL in some patients receiving class IC antiarrhythmic agents, this does not explain why there is such a high rate of success in maintaining sinus rhythm after CTI ablation with continued administration of an AAD. Several explanations for this phenomenon can be considered. We suggest that the most compelling mechanism is that conversion from AF to typical AFL upon the initiation of class Ic antiarrhythmics selects for a subset of patients who are more likely to respond to the medication itself. Alternatively, this may be an incidental finding. Class Ic antiarrhythmics are already effective agents in maintaining sinus rhythm, as described above. It has also been shown that CTI ablation alone can sometimes reduce the incidence of further AF.^37^ The combined use of both modalities may simply have an additive effect on the maintenance of sinus rhythm. We find this to be a less-compelling explanation, however, given the magnitude of the difference in efficacy between the hybrid approach and the effect of the sum of individual approaches.^6^ Given the small size of studies reporting on use of this combined approach for rhythm management in AF, further investigation is still needed to fully describe the reasons for the high efficacy of this technique. Should findings from such research support our primary hypothesis that conversion from AF to AFL using class Ic agents selects for patients more likely to respond to the medication, it would be interesting to then determine whether or not different subsets of patients are more likely to respond to different antiarrhythmics. Ultimately, such an investigation could lead to the discovery of clinical or anatomic factors that could help to guide the choice of antiarrhythmic agent. We suggest that studies should be focused on the ability of various medications to promote the formation of a functional line of block along the crista terminalis.

## Conclusion

AF and AFL are highly related arrhythmias. It has long been noted that class Ic antiarrhythmic agents can promote the conversion of AF to AFL. This phenomenon occurs because rapid atrial rates during AF result in the formation of a functional line of block along the crista terminalis, creating a substrate for the macroreentrant circuit of AFL. AADs may decrease the threshold for the formation of this line of block, enabling it to be stable at slower atrial rates and, therefore, capable of being maintained by the AFL circuit itself. A hybrid approach to the treatment of AF has been suggested that involves CTI ablation and the maintenance of the antiarrhythmic medication for patients who initially convert from AF to typical AFL. This strategy has been shown to have a high success rate in limited studies. The reason for this high efficacy is unknown. We suggest that conversion from AF to AFL with class IC antiarrhythmics selects for a subset of the population in whom these medications are highly effective. Understanding this phenomenon may lead to better medication selection for patients or the development of more effective antiarrhythmic medications in the future. We also recommend that this scenario should not necessarily be viewed as a failure of the AAD. Despite advances in ablation technology, PVI remains a more invasive procedure with higher risks than isolated CTI ablation. While there is not yet enough evidence to recommend routinely omitting PVI in these patients, we feel that, in select patients, a trial of CTI ablation without PVI plus continuation of the current AAD is warranted and may result in fewer complications.

## Figures and Tables

**Table 1:** Studies Investigating the Efficacy of a Hybrid Approach for Rhythm Management

Study	Sample Size and Details of Provoked Flutter	Baseline Patient Characteristics	Antiarrhythmic Medication(s)	Outcomes	Method of Monitoring	Length of Monitoring
Schumacher et al.^2^	24 patients (20 with typical AFL on EPS, 19 with successful ablation) 20 patients who did not develop AFL used as controls	Paroxysmal AF patients from registry	Flecainide Propafenone	7/19 without recurrence of AF after AFL ablation 8/19 had significantly lower recurrences per year than before ablation (2.8 ± 1.6 vs. 21 ± 24; p < 0.001) 4/19 had no benefit after ablation Significantly fewer recurrences per year were seen in the ablation group than in the control group (2.7 ± 3.6 vs. 7.8 ± 9.2; p < 0.05) Significantly fewer recurrences per year were seen in the ablation group after therapy versus before therapy (2.7 ± 3.6 vs. 10.2 ± 5.4; p < 0.001)—this decrease was greater than in the control group but was not statistically evaluated	Ambulatory Holter monitoring at 1, 3, 6, and 12 months after discharge Monthly questionnaires	11 ± 4 months
Nabar et al.^3^	24 patients (13 with typical AFL, 8 with atypical AFL, 3 with coarse AF; 1/13 with typical AFL had Ebstein anomaly and unsuccessful ablation)	Symptomatic recurrent AF 9/24 paroxysmal 15/24 persistent	Flecainide Propafenone	11/13 with typical AFL without further AF recurrences (2 stopped AADs, 2 started on sotalol) 4/8 with atypical flutter had a reduction in frequency of recurrences (frequency not reported) 0/3 with coarse AF had any change in AF frequency	History, ECG, and Holter recordings at 8 weeks and then at 3-month intervals 4/24 patients had cardiovascular implantable electronic devices, which were also used to document recurrence	13 ± 6 months
Huang et al.^4^	13 patients (11 with typical flutter, 2 with atypical flutter) 9 patients with isthmus-dependent flutter on EPS with successful ablation 4 patients with complex activation pattern and no successful ablations)	Symptomatic AF 11/13 paroxysmal AF 1/13 chronic AF 1/13 paroxysmal but AF and AFL	Amiodarone Flecainide Propafenone Quinidine β-blocker only	8/9 without recurrence	Clinic visits, telephone interviews, and review of the medical records and ECGs obtained inpatient or outpatient after ablation	14.4 ± 6.9 months
Nabar et al.^5^	14 patients (all with typical flutter) 12/14 were successfully ablated, 1/14 had partially successful ablation but bidirectional block not demonstrated, and 1/14 had Ebstein anomaly and unsuccessful ablation	Drug-resistant symptomatic AF	Flecainide Propafenone	9/13 remained symptom-free (1 of whom started amiodarone and 1 of whom switched to atenolol) 2/13 had few, short-lasting, well-tolerated episodes but quantification of preintervention episode frequency is not available for comparison 2/13 patients remained symptomatic No quantification of asymptomatic recurrences was provided	Follow up at 8 weeks and then 3-month intervals Holter recordings at predischarge, 8 weeks, and 12 weeks after ablation and for any symptoms suggesting arrhythmia Phone interview at end of study	4 months; range: 2–13 months
Stabile et al.^6^	71 patients with conversion from AF to typical AFL upon infusion of intravenous flecainide randomized to: oral AAD (A), hybrid therapy (B), or ablation only (C) 37 patients without conversion upon infusion of flecainide were designated as the control group (D) and received hybrid therapy	Drug-refractory paroxysmal or chronic AF	Flecainide	Group A: 78% recurrence Group B: 42% recurrence Group C: 92% recurrence Group D: 92% recurrence Group B demonstrated statistically fewer recurrences versus all other groups No statistical difference in recurrence was found between other groups	Monthly clinical exam and ECG ECG for any symptoms	24 ± 7.2 months

AAD: antiarrhythmic drug; AF: atrial fibrillation; AFL: atrial flutter; ECG: electrocardiogram; EPS: electrophysiology study.
